# Assessment of acquisition protocols for routine imaging of Y-90 using PET/CT

**DOI:** 10.1186/2191-219X-3-11

**Published:** 2013-02-17

**Authors:** Thomas Carlier, Thomas Eugène, Caroline Bodet-Milin, Etienne Garin, Catherine Ansquer, Caroline Rousseau, Ludovic Ferrer, Jacques Barbet, Frédéric Schoenahl, Françoise Kraeber-Bodéré

**Affiliations:** 1 Nuclear Medicine Department, University Hospital of Nantes, Place Alexis Ricordeau, Nantes, 44093, France; 2 Nantes-Angers Cancer Research Center, UMR 892 INSERM and UMR 6299 CNRS, 8 quai Moncousu BP 70721, Nantes, 44007, France; 3 Nuclear Medicine Department, Cancer Center, Rue de la Bataille Flandres Dunkerque, Rennes, 35042, France; 4 Nuclear Medicine Department, Integrated Center of Oncology, Boulevard Jacques Monod, Saint-Herblain, 44805, France; 5 Molecular Imaging, Siemens Healthcare, Hartmnannstrasse 16, Erlangen, 91052, Germany

**Keywords:** 90-Y, PET, Quantification, Detectability, Radioembolization

## Abstract

**Background:**

Despite the early theoretical prediction of the 0^+^-0^+^ transition of ^90^Zr, ^90^Y-PET underwent only recently a growing interest for the development of imaging radioembolization of liver tumors. The aim of this work was to determine the minimum detectable activity (MDA) of ^90^Y by PET imaging and the impact of time-of-flight (TOF) reconstruction on detectability and quantitative accuracy according to the lesion size.

**Methods:**

The study was conducted using a Siemens Biograph® mCT with a 22 cm large axial field of view. An IEC torso-shaped phantom containing five coplanar spheres was uniformly filled to achieve sphere-to-background ratios of 40:1. The phantom was imaged nine times in 14 days over 30 min. Sinograms were reconstructed with and without TOF information. A contrast-to-noise ratio (CNR) index was calculated using the Rose criterion, taking partial volume effects into account. The impact of reconstruction parameters on quantification accuracy, detectability, and spatial localization of the signal was investigated. Finally, six patients with hepatocellular carcinoma and four patients included in different ^90^Y-based radioimmunotherapy protocols were enrolled for the evaluation of the imaging parameters in a clinical situation.

**Results:**

The highest CNR was achieved with one iteration for both TOF and non-TOF reconstructions. The MDA, however, was found to be lower with TOF than with non-TOF reconstruction. There was no gain by adding TOF information in terms of CNR for concentrations higher than 2 to 3 MBq mL^−1^, except for infra-centimetric lesions. Recovered activity was highly underestimated when a single iteration or non-TOF reconstruction was used (10% to 150% less depending on the lesion size). The MDA was estimated at 1 MBq mL^−1^ for a TOF reconstruction and infra-centimetric lesions. Images from patients treated with microspheres were clinically relevant, unlike those of patients who received systemic injections of ^90^Y.

**Conclusions:**

Only one iteration and TOF were necessary to achieve an MDA around 1 MBq mL^−1^ and the most accurate localization of lesions. For precise quantification, at least three iterations gave the best performance, using TOF reconstruction and keeping an MDA of roughly 1 MBq mL^−1^. One and three iterations were mandatory to prevent false positive results for quantitative analysis of clinical data.

**Trial registration:**

http://IDRCB 2011-A00043-38 P101103

## Background

Yttrium-90 (half-life, 64 h) is a radionuclide used in targeted radionuclide therapy, particularly for radioimmunotherapy (RAIT), peptide receptor radionuclide therapy (PRRT), and selective internal radiotherapy (SIRT). Promising clinical results have been obtained for the treatment of B-cell lymphomas with anti-CD20 and anti-CD22 monoclonal antibodies [1,2], while the treatment of neuroendocrine tumors with somatostatin analogues has proven its benefit in the last decade [3,4]. One RAIT product labeled with ^90^Y has been already approved by the regulatory authorities (Zevalin®, Bayer Corporation, Pittsburgh, USA). Encasing ^90^Y in a resin or a glass sphere has also provided a promising approach for the treatment of hepatocellular carcinoma (HCC) and unresectable liver metastases with SIRT [5-7]. Two microsphere products are approved and available for clinical practice: TheraSphere® (glass microspheres; MDS Nordion, Ontario, Canada) and SIR-Spheres® (resin microspheres; Sirtex Medical, North Sydney, Australia).

The emission spectrum of ^90^Y is almost exclusively β^−^ (mean energy 933.6 keV), making the irradiation of macroscopic tumoral lesions possible. However, despite the increasing therapeutic applications of ^90^Y, imaging the distribution of this isotope in the patient's body remains a significant challenge when assessing the quality of tumor targeting after injection, as well as when performing systematic dosimetry studies. While pre-therapeutic imaging with surrogate tracers remains the best and only means of adapting the therapeutic activity to be injected (such as ^111^In or ^68^ Ga for RAIT or ^99m^Tc for SIRT), it could pose significant organizational issues depending on local constraints (especially for ^111^In pre-therapeutic studies) as well as additional costs. Moreover, the biodistribution of a radiopharmaceutical labeled with a different radionuclide may imperfectly predict that of ^90^Y-labeled radiopharmaceuticals [8]. Therefore, *in vivo* imaging during the treatment phase can predict toxicity and efficacy, and can help optimize the injected activity in subsequent courses, given that radiotherapy treatments usually require repeat injections.

The current clinical practice for *in vivo* imaging of such treatment involves a SPECT acquisition using bremsstrahlung and remains the method of choice to assess the distribution of ^90^Y-labeled radiopharmaceuticals [9]. Although reconstruction algorithms suitable for bremsstrahlung imaging of ^90^Y are under development [9,10], the resulting images have the disadvantage of having a low spatial resolution and poor quantification performance, especially for small lesions when using parallel-hole collimators. However, a recent study showed the benefits of a using pinhole collimator for bremsstrahlung imaging of ^90^Y with very promising results [11].

The prediction and discovery of the 0^+^-0^+^ transition of ^90^Zr [12], which results in a β^+^/β^−^ pair creation with a very low branching ratio of 31.86 × 10^−6^[13], provide the opportunity to detect ^90^Y distribution using PET images [14]. The first clinical applications of PET imaging with ^90^Y have been tested recently in the context of radioembolization [15,16], PRRT [17], and RAIT for lymphoma [18]. These studies focused primarily on the detection of lesions incorporating a very large amount of tracer (typically several MBq mL^−1^, equivalent to several tens of Bq mL^−1^ when considering the pair creation), thus facilitating the visualization and accurate measurement of radioactive concentration. Similarly, the value of using time of flight (TOF) information for this type of acquisition in this context of high radioactive concentration was recently investigated [19]. In a RAIT protocol, the expected concentration in the lesions generally ranges from 0.01% or less to 1% of the total injected activity. This leads to a typical concentration of several tens of kBq mL^−1^[20].The aim of this work was three-fold. The first objective was to determine the minimum detectable activity (MDA) with PET imaging using ^90^Y for a LSO-based acquisition system in relation to lesion size. The second objective was to study the impact of TOF reconstruction on detectability and quantitative accuracy according to lesion size and finally to correlate these results with analysis on patient data.

## Methods

### PET/CT system

The whole study was conducted using the PET/CT Biograph® mCT 40 (Siemens Healthcare Molecular Imaging, Hoffman Estates, IL, USA). The patient bore aperture was 78 cm (inner diameter 84.2 cm) with an axial field of view (FOV) of 22 cm. The system was equipped with 32,448 LSO crystals (4 × 4 × 20 mm^3^). The detection energy window was set to 435 to 650 keV for a coincidence window of 4.1 ns. The system was able to record TOF information between two events in coincidence. Given the sufficient count rate capacity of the system [21] confirmed by in-house performance measurements of count losses according to the NEMA NU 2–2007 methods [22], it was chosen to not incorporate a copper ring between the source and the LSO crystals to lower the count rate originating from the bremsstrahlung [19,23].

### Phantom study

#### Source preparation and acquisition protocol

A standard IEC/NEMA 2001 torso-shaped phantom (PTW Freiburg GmbH, Freiburg, Germany) was used with five spheres (internal diameters 10, 13, 17, 22, and 28 mm). The spheres were initially filled with free ^90^Y (8100 ± 5% kBq mL^−1^). The phantom volume (9.1 L) was also filled uniformly with an initial activity of ^90^Y (200 ± 5% kBq mL^−1^) to simulate a sphere-to-background ratio (SBR) of approximately 40:1. This SBR was chosen to simulate a high tumor uptake relative to the background typically encountered in a RAIT procedure. Acquisition of the phantom was performed nine times over 14 days, starting from an initial activity concentration in the spheres of 6,740 kBq mL^−1^ decreasing to 490 kBq mL^−1^. All measurements were performed over a duration of 30 min using a single-bed position including the whole phantom in the FOV.

#### Reconstruction

The acquisitions were reconstructed with the proprietary implementation of the point spread function (PSF) ordinary Poisson (OP) ordered subset expectation maximization (3D OP-OSEM PSF) available for this scanner [24]. We have used both TOF and non-TOF reconstructions with respectively 21 and 24 subsets, which are the clinical default settings. One or three iterations were used for both TOF and non-TOF reconstructions to assess the impact of increasing the number of iterations on the sphere detection and quantification accuracies. A maximum of three iterations was set in order to limit noise amplification. Additional parameters such as the impact of two iterations were omitted as the study was limited to evaluating the trade-off between detectability and quantification. Datasets were reconstructed into standard 200 × 200 × 109 matrix size using a 4 × 4 × 2 mm^3^ voxel size. A 3D Gaussian post-smoothing of 2 mm full-width at half maximum (FWHM) was applied. To allow direct comparison, all reconstructed images were coregistered to the image corresponding to the first acquisition. This was facilitated by placing the phantom at identical axial and transaxial positions using laser lights of the system and markings made on the phantom.

### Data processing

#### Detectability

The detectability was assessed using a contrast-to-noise ratio (CNR) based figure of merit (FOM), and its formalism is given below. As mentioned before, the branching ratio of internal pair creation for ^90^Zr is remarkably small compared to usual PET tracers. The detection of lesions incorporating ^90^Y, therefore, is challenged by very low levels of radioactive distribution, or signal. In this respect, the determination of a MDA has been the subject of several studies in preclinical situations [25,26]. The MDA may be evaluated using the Rose criterion [27]. It states that an object is discernable when CNR > 5, with:

(1) $$\mathrm{CNR}=\frac{C_{L-}C_{B}}{\sigma_{B}},$$

where *C*_L_, *C*_B_, and *σ*_B_ are the lesion, the background, and the background noise intensities, respectively.

The background noise $\sigma_{B}^{r}$ measured inside a given region of interest (ROI) *R*_r_ of *V* voxels of a given slice was defined as:

(2) $$\sigma_{B}^{r}=\sqrt{\frac{1}{V}\sum_{j\in R_{r}}{\left({\hat{m}}_{r}-f_{j}\right)}^{2}}.$$

Here, ${\hat{m}}_{r}$ is the average of activity for the *V* voxels inside the ROI *R*_r_ in the reconstructed image *f*. The final noise FOM was measured in *R* ROIs in the background of a given slice, and was defined as:

(3) $$\sigma_{B}=\frac{1}{R}\sum_{r=1}^{R}\sigma_{B}^{r}.$$

The *R* = 20 ROIs localized in the background were randomly chosen, in such a way that they were separated by at least two voxels from each other and at least three voxels from the phantom border. Each ROI consisted of *V* = 32 voxels. The transverse slice aligned with all sphere centers was chosen for analysis.

This FOM proved to be equivalent to a rigorous noise assessment using multiple statistically independent replicates while avoiding the correlation between voxels that results from iterative reconstruction [28].

The relation (1) is established for a signal present in a single voxel. However, it can be extended to a lesion covering *N* voxels [25] using the equation:

(4) $$\mathrm{CNR}=\frac{C_{L}-C_{B}}{\sigma_{B}}\times \sqrt{N}\times \mathrm{PVE},$$

where PVE is the partial volume effect calculated for each sphere. The PVE was evaluated with an ^18^ F-based acquisition with similar phantom preparation, by drawing a circular ROI with an internal diameter equal to the actual diameter of the spheres. For this setup, the SBR was chosen identical to that of the ^90^Y-based acquisitions. The measurement was repeated 30 times to supply 30 independent datasets. The mean PVE was derived from these 30 datasets.

As the spheres were close to a circular-shaped object, we chose to modify the limit of detectability for CNR according to a previous work based on human observers [29]. Although determined in the different context of simulated noisy micrographs, they have suggested that circular-shaped object was detected if CNR > 8 for an area equivalent to those considered in the present work. Thus, this limit will be considered in this work.

#### Recovered activity in the spheres

The total activity in each sphere was calculated for each of the nine ^90^Y-based acquisitions and was compared to the theoretical value as a percentage of recovered activity. The total activity in a sphere was computed as the mean intensity of all voxels within the volume of interest (VOI) corresponding to the sphere. The VOI for each sphere was determined from the sum image of the 30 ^18^ F-based independent acquisitions mentioned earlier for PVE determination. The five VOIs were calculated using a threshold relative to the maximum value in the VOI. The intensity threshold was chosen to obtain the smallest difference between the true volume and the measured volume.

#### Spatial distribution of the signal in spheres

The reconstruction of a very weak signal can be significantly biased when assessing its spatial distribution. The change in the distribution of the signal within the spheres according to the radioactive concentration was evaluated by calculating the root mean square error (RMSE) using the following equation:

(5) $$\mathrm{RMSE}\left(S^{90Y}{,S}^{18F}\right)=\sqrt{\frac{1}{N}\sum_{k=1}^{N}{\left(B_{k}^{S^{90Y}}-B_{k}^{S^{18F}}\right)}^{2}},$$

where $B_{k}^{S^{90Y}}$ denotes the contents of voxel *k* in a sphere for the yttrium-based acquisition, and $B_{k}^{S^{18F}}$ represents fluorine-based acquisition. The signal considered for the fluorine-based acquisition was measured from a mean image computed from the 30 independent acquisitions described in the ‘Recovered activity in the spheres’ section. For the RMSE calculation, the images were first normalized by the mean signal for each acquisition and for each sphere considered.

#### Count rate

The acquisition of a weak signal may be masked by natural radioactivity of ^176^Lu in LSO crystals (about 2.59% of the lutetium element). The background signal generated by ^176^Lu can contribute to the amount of random and true coincidences [26,30]. For each acquisition, prompt and random coincidence rates were measured. A long acquisition (approximately 900,000 registered true coincidences) with no activity present in the field of view was performed to determine the ^176^Lu background count rate.

### Patient study

A clinical study was performed on ten patients, including six patients with HCC treated by hepatic SIRT using TheraSphere® (MDS Nordion) or SIR-Spheres® (Sirtex Medical), and four patients with B lymphoma treated using anti-CD20 Zevalin® (Bayer) or anti-CD22 epratuzumab (Immunomedics, Inc., Morris Plain, NJ, USA) ^90^Y-based RAIT. Among the six HCC patients, five had macroscopic lesions, and one had a diffuse liver disease. Among the four lymphoma patients, two had a diffuse large B-cell lymphoma, and one had a follicular lymphoma receiving RAIT as consolidation treatment after an induction therapy. The other patient had a follicular lymphoma and was undergoing first-line treatment with Zevalin® (Bayer). All patients underwent a 30-min PET imaging session with all acquisition parameters identical to those of the phantom study. Reconstructions were performed with or without TOF information with one or three iterations. For those who underwent radioembolization, the total amount of reconstructed activity was compared to the theoretical activity injected to the patient, as measured by the dose calibrator before injection.

All patients gave informed written consent in accordance with institutional guidelines, including the declaration of Helsinki. The trials were approved by the responsible ethics committee.

## Results

### Detectability and minimum detectable activity

**Figure 1:**
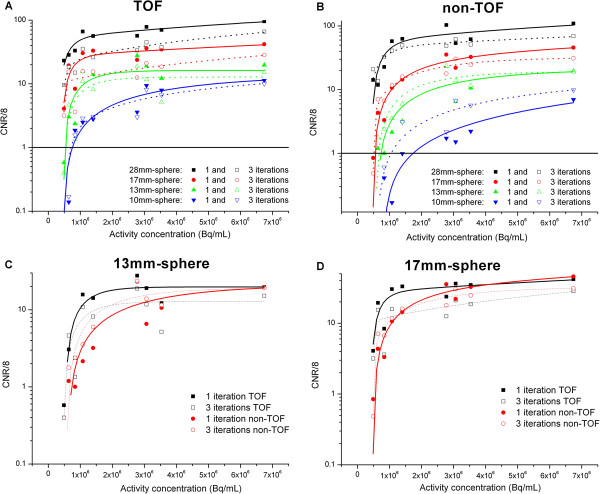
**CNR normalized by the minimum CNR value.** CNR normalized by the minimum CNR value (8) according to the activity concentration in all spheres for TOF reconstruction (**A**), non-TOF reconstruction (**B**), the 13-mm diameter (**C**), and the 17-mm diameter sphere (**D**). The thick line ( in panels **A** and **B**) is the minimum normalized CNR value (1) above which the detectability could be considered as true. Experimental data points were fitted to highlight the relationship between CNR and activity concentration.

Figure 1 shows the detectability performance on phantom for both TOF and non-TOF reconstructions and one or three iterations. When reconstruction was made with TOF, higher detectability was reached with a small number of iterations (one iteration with 21 subsets), regardless of the sphere size. This result held true for non-TOF reconstructions with a less marked difference between one and three iterations. All spheres with a diameter greater than 17 mm were detected regardless of the radioactive concentration in the spheres considered in this study for TOF reconstruction (22 mm for non-TOF).

The MDA for the smaller volume was about 1 MBq mL^−1^ for TOF reconstructions (and 3 MBq mL^−1^ for non-TOF) when using reconstructions with a single iteration. Despite an absolute value greater than 1 for the normalized CNR, determining the MDA for this smallest sphere reconstructed without TOF was particularly difficult. This was due to the slow decrease in the CNR with the radioactive concentration, as well as the high noise in the background discussed above. Consequently, a comparison between the qualitative analysis of reconstructed images and the quantitative values (CNR) was taken into account to calculate the actual MDA for the smallest sphere and the non-TOF reconstruction case.

The benefit of adding TOF information is illustrated in Figure 1 for spheres of diameters 17 and 13 mm. Taking into account statistical fluctuations, we observed no significant gain in using TOF for small structures more than 17 mm in diameter and concentrations greater than approximately 2 to 3 MBq mL^−1^ when using one iteration for the CNR figure of merit. As expected, smaller structures lead to an improved MDA for TOF reconstructions regardless of the activity concentration.

**Figure 2:**
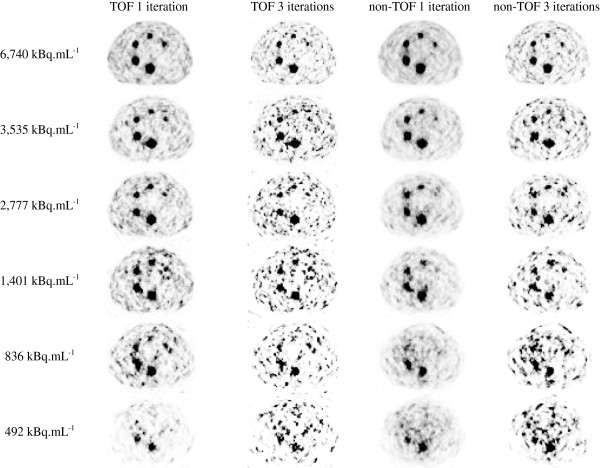
**Central slice of the phantom.** Transverse slice passing through the middle of the spheres for TOF and non-TOF reconstructions as a function of activity concentration in the spheres. The background gray scale is identical for all images.

Figure 2 shows the slice crossing all spheres at their largest diameter for the nine acquisitions performed. All spheres remain clearly visible down to a concentration of 1 MBq mL^−1^ for TOF reconstructions with one iteration. For the same number of iterations, the smallest sphere (10 mm in diameter) was no longer visible below a concentration of 3 MBq mL^−1^. In general, the change from one to three iterations substantially increased the noise in the reconstructed images and made visual detection difficult for radioactive concentrations below 3 MBq mL^−1^.

### Recovered activity in the spheres

**Figure 3:**
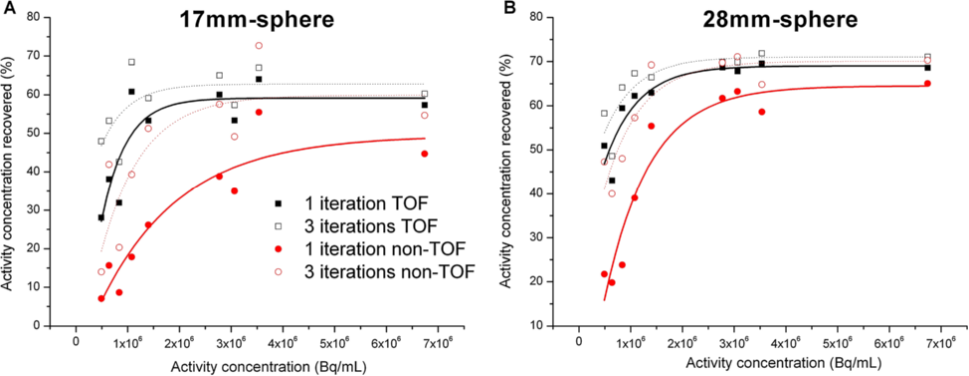
**Recovered activity.** Percentage of recovered activity according to the activity concentration for the 17-mm diameter (**A**) and 28-mm diameter spheres (**B**). Experimental data points were fitted to highlight the relationship between recovered activity and activity concentration.

Figure 3 shows the percentage of activity reconstructed for the two spheres of 17 and 28 mm only. The trend for reconstructed activities in relation to the activity concentration within spheres was similar for all spheres. When the TOF information was used during reconstruction, one iteration gave a slight underestimation (less than 5%) compared to the results obtained with three iterations, except for the activity concentrations below 1 MBq mL^−1^ where the underestimation was greater than 10%. The non-TOF reconstructions performed with a single iteration underestimated the activity concentration in the spheres by between 10% and 20% compared to TOF reconstruction with three iterations for large structures (more than 22 mm in diameter) when the activity concentration was greater than 3 MBq mL^−1^. This underestimation reached more than a factor of 2 for small structures (less than 17 mm in diameter) or concentrations below 1 MBq mL^−1^.

Finally, TOF reconstructions with one iteration and non-TOF with three iterations gave very similar results.

### Spatial distribution of the signal in spheres

**Figure 4:**
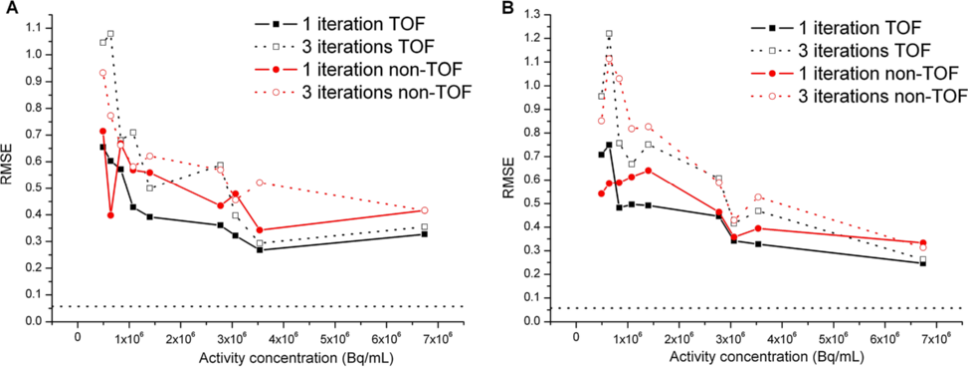
**Root mean square error.** RMSE according to the activity concentration for the 17-mm diameter (**A**) and 28-mm diameter (**B**) spheres. The two dotted lines represent the minimal RMSE calculated with three independent ^18^ F-based acquisitions.

Figure 4 shows the RMSE according to activity concentration for both spheres of 17 and 28 mm in diameter. The conclusions were equivalent for other spheres.

The mean RMSE calculated based on three independent ^18^ F-based acquisitions is represented by a dotted line in order to estimate the minimum value achievable for the RMSE. As suggested by the images (Figure 2), a lower radioactive concentration led to a higher RMSE. This resulted in substantial errors for radioactive concentrations of less than about 1 MBq mL^−1^. Again, TOF reconstruction with one iteration gave results closest to the expected spatial distribution regardless of the radioactive concentration for a homogeneous activity distribution.

### Count rate

**Figure 5:**
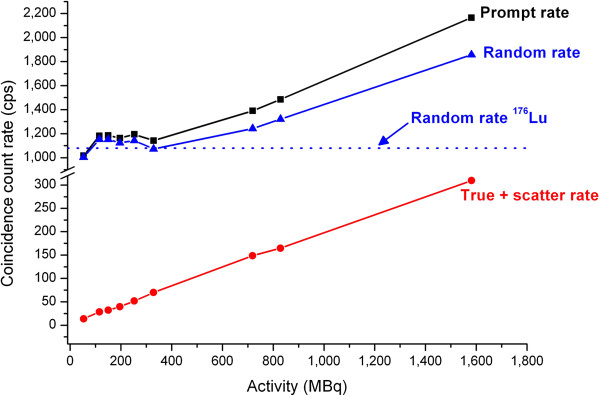
**Count rate.** Count rate according to total activity in the field of view of the scanner. The blue dotted line shows the natural ^176^Lu count rate for random coincidences.

Despite the extended field of view with a large acceptance of 13.2° for incoming single events, no saturation of the detectors was observed, which is in accordance with previous studies using similar systems [19]. Figure 5 shows the count rate of prompt, delayed, and true coincidences for the total activity (including background and spheres). The dotted horizontal line shows the count rate of delayed coincidences due to the contribution of ^176^Lu. The count rate of true coincidences when no source was located in the field of view was 5 cps for the default acquisition setup of the scanner. The total activity of ^176^Lu contained in the 32,448 crystals of the Biograph® mCT (Siemens AG, Munich, Germany) was estimated at about 2.6 MBq. The total useful signal (prompt coincidences) was still significantly greater than the residual ^176^Lu background noise for activities exceeding 400 MBq in the field of view (greater than one standard deviation of the Poisson noise). As a comparison, the ratio between the prompt and random coincidence rates for a standard ^18^ F-FDG scan was approximately 3 for a step centered on the liver. This ratio was roughly 1.2 for 1.5 GBq of ^90^Y centered in the FOV. This highlights the importance of having an unbiased estimate of random coincidences for low count rates such as those typically encountered for ^90^Y imaging [30].

### Patient results

**Figure 6:**
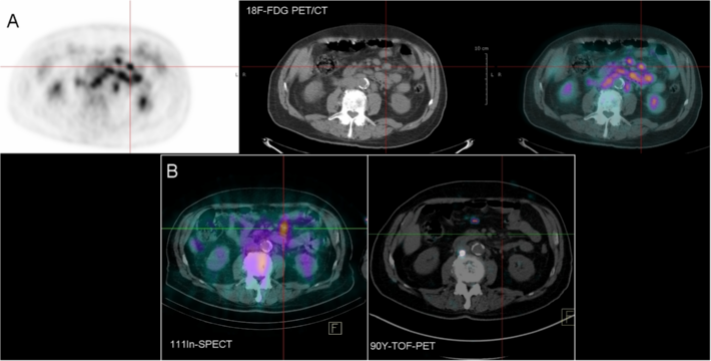
^**18 **^**F-FDG PET and immuno-PET.** Patient with follicular lymphoma injected with ^111^In- and ^90^Y-Zevalin® (Bayer) 7 days apart. (**A**) Pre-therapeutic scan after injection with ^18^ F-FDG and (**B**) immunoscintigraphy recorded 6 days (bottom left) after ^111^In-Zevalin® (Bayer) injection showed an abnormal focus corresponding to a lymphoma mass detected on the pre-RAIT CT and ^18^ F-FDG PET (**A**). No relevant signal was detected by ^90^Y-PET (bottom right).

**Figure 7:**
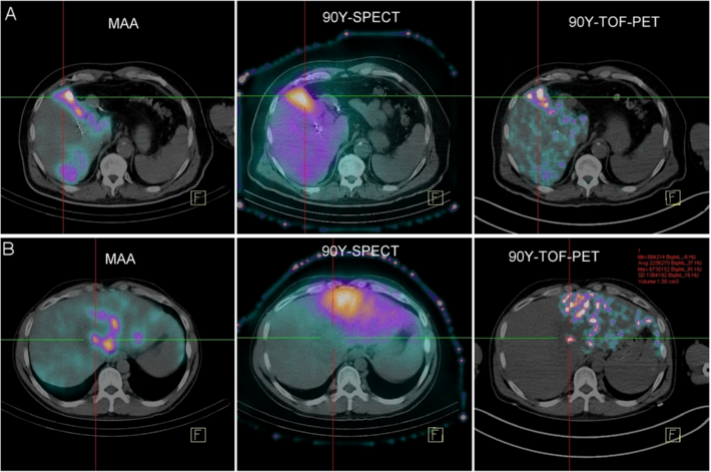
**Radioembolization images.** Different imaging sessions for two patients (**A** and **B**) with HCC: ^99m^Tc-MAA SPECT (left), bremsstrahlung SPECT (center), and ^90^Y-PET (right). The reference lines cross a tumor lesion for the two patients. Imaging sessions for the second patient (**B**) show a clear discrepancy between pre-therapeutic (left) and post-therapeutic (right) imaging sessions. Tumor uptake was detected with ^90^Y-PET (right) but not with bremsstrahlung SPECT (center). Measurement of the tumor lesion for patient B is illustrated in red (right).

No ^90^Y uptake was detected using PET in the four lymphoma patients. Three of them had small tumor masses after the induction treatment (Figure 6), and one had a diffuse cutaneous disease but no masses. For the six HCC patients, high radioactive concentration in the tumors was detected using ^90^Y-PET, distinguishing tumors of different sizes. The benefit of performing an ^90^Y-PET acquisition in the context of HCC disease is illustrated in Figure 7 for two different patients. One patient showed a good agreement between the ^99m^Tc-labeled macroaggregates (^99m^Tc-MAA) SPECT scan, the bremsstrahlung SPECT scan, and the ^90^Y-PET acquisition. The other exhibited a clear discrepancy between the pre-treatment ^99m^Tc-MAA SPECT scan and post-therapeutic acquisitions due to a slight difference between injection techniques and an asymmetric arterial flow. No tumor uptake was detected with the bremsstrahlung image, while ^90^Y-PET imaging clearly highlighted a small uptake by the tumor. This example clearly highlights the benefit of using ^90^Y-PET for small lesions compared to bremsstrahlung imaging. Moreover, the activity concentration for this small tumor (1.5 cm^3^) was measured with one iteration and TOF (1.1 MBq mL^−1^), three iterations and non-TOF (1.3 MBq mL^−1^), and three iterations and TOF (2.2 MBq mL^−1^) reconstructions. In this case, the difference could reach a factor 2 between the different sets of reconstruction parameters*.* Finally, for this patient, the ^90^Y-PET was used to calculate the dose absorbed by the normal liver in order to plan a second hepatic SIRT, more selective for the tumor. A good agreement between ^99m^Tc-MAA, bremsstrahlung, and ^90^Y-PET imaging sessions was found for the four remaining patients.

Table 1 gives the total amount of activity in the liver for the six patients and several reconstruction parameters in light of theoretical injected activity as measured by the dose calibrator. Measurement of initial activity was highly unreliable [31] and was very difficult for the residual activity measurement. This was illustrated by the results shown in Table 1 in which the reconstructed activity was overestimated for one patient and underestimated for the others, if one considers the correct activity to be that measured by the dose calibrator.

**Table 1:** Reconstructed activity

**Patient**	**Number of iterations**			**Measured activity before injection**
	**1 (TOF)**	**3 (TOF)**	**3 (nTOF)**	
1	2401 (+23%)	2515 (+28%)	2164 (+11%)	1958
2	1194 (−10%)	1374 (+3%)	1231 (−8%)	1332
3	468 (−9%)	534 (+4%)	428 (−17%)	514
4	324 (−23%)	361 (−14%)	348 (−17%)	422
5	1037 (−8%)	1090 (−3%)	1009 (−10%)	1124
6	638 (−19%)	721 (−8%)	659 (−16%)	784

Total reconstructed activity (MBq) for the six patients with HCC according to reconstruction parameters. The relative differences between reconstructed activity and activity measured before injection are in parentheses.

## Discussion

This work addresses one aspect of the general question of PET imaging with very low activity, such as that encountered typically in PET monitoring of therapeutic ion irradiation [32]. Here, we showed that although ^90^Y imaging is possible, it is useful only for a size and concentration above a given level of radioactive concentration and when optimized parameters are implemented.

Previous work in the area of ^90^Y imaging by PET has established this technique as being potentially useful for the precise assessment of the spatial distribution of microspheres and to determine the quantitative input activity map for subsequent dosimetric calculations [15,17]. The advantage of using TOF vs. non-TOF reconstruction has also been recently studied in a comprehensive manner [19]. However, all these studies were conducted in the context of therapeutic radioembolization with microspheres [15,19] or PRRT [17]. This type of treatment results in a radioactive concentration of several MBq mL^−1^ or even tens of MBq mL^−1^[17-19]. To our knowledge, no studies have reported the calculation of the minimum detectable radioactive concentration of ^90^Y by PET imaging, or its relationship to lesion size and its impact on the quantification of the signal. The current work was conducted in the context of a systemic injection when the radioactive concentrations are much smaller than SIRT, the ratio of lesion to background is, in theory, high and the signal from the natural radioactivity of ^176^Lu is of the same order of magnitude as that obtained for low concentrations of ^90^Y.

The experimental data allowed us to determine some practical aspects regarding the MDA or the influence of reconstruction parameters on detectability and quantification accuracy. Mainly, for lesions with an activity concentration exceeding 2 to 3 MBq mL^−1^, the best detectability was provided by reconstruction using TOF or non-TOF information with one iteration whatever the lesion size. The TOF information and one iteration were required for an activity concentration below 2 to 3 MBq mL^−1^ regardless of the lesion size, in terms of best detectability. These results are consistent with those reported previously in a context where the signal to background ratio was one order of magnitude lower [19] than in our work. Table 2 summarizes the estimated MDA for all lesion sizes, showing that the best detectability was effectively reached with one iteration (TOF or non-TOF). Regarding the accuracy of quantitative information, the best compromise was reached using TOF information and three iterations regardless of the radioactive concentration and lesion size.

**Table 2:** Minimum detectable activity

**Lesion size (mm)**	**TOF reconstruction (kBq mL**^**−1**^**)**	**non-TOF reconstruction (kBq mL**^**−1**^**)**
28	<500	<500
22	<500	<500
17	≈600	≈1,000
13	≈900	≈1,400
10	≈1,000	≈3,000

MDA for the different sphere and TOF reconstruction option.

These experimental findings were compared to those derived in clinical situation and allowed a comprehensive knowledge of the signal collected in patients.

We must emphasize that the signal from the ^176^Lu became a significant contribution for random coincidences for radioactive concentration below 1 MBq mL^−1^ in a cold background (or with a high ratio between the signal in the background and the spheres). After reconstruction, the residual signal resulted in multiple isolated foci of moderate intensity in the reconstructed volume that may increase the number of false positive results when small lesions or low concentrations are involved. This reconstructed signal is further amplified by the necessary attenuation correction and appears more intense in the reconstructed volume, especially when three iterations are used. In this last case, the radioactive concentration of a false positive could be in the range of few MBq mL^−1^ due to statistical fluctuations. Generally, when the concentration measured in a lesion extracted from a volume reconstructed with one iteration and TOF is greater than 1 MBq mL^−1^, the lesion could be considered as true positive even if the concentration measured in a volume reconstructed with TOF and three iterations is in the range of few MBq mL^−1^. Conversely, a concentration measured as high as few MBq mL^−1^ for a reconstructed volume with TOF and three iterations but with an intensity of less than few hundred of kBq mL^−1^, when the volume is reconstructed with one iteration and TOF, could be considered as false positive. This effect was clearly observed in the phantom measurements. This is a potential method to discriminate true positive from false positive in the context of clinical ^90^Y-PET imaging, but it still needs to be confirmed on larger clinical datasets by comparing it with ^99m^Tc-MAA and contrast-enhanced CT. Finally, unlike the hypothesis provided by Campbell et al. [33] and van Elmbt et al. [19], we found a mean tumor-to-liver uptake ratio of roughly 10 for patients with HCC. This led to higher mean activity concentrations of 10 MBq mL^−1^ in the tumor and approximately 900 kBq mL^−1^ (range 200 to 1,300 kBq mL^−1^) in the liver, higher than those reported in the latter studies.

One of the main limitations of our study was the estimated CNR based on the assumption of uncorrelated noise and was generally assessed for data reconstructed with linear algorithms such as FBP. The use of 3D OP-OSEM and a PSF model within the chosen reconstruction method led to a correlation between voxels, which may have affected the noise estimation. However, based on the chosen parameters, we assumed that this effect would be limited to the extent of 2 to 3 voxels only [34], despite the post-smoothing Gaussian function (2 mm FWHM) and could be neglected in this work. Moreover, the definition of the limit of detectability of lesions (i.e., CNR > 8) is a complex function depending on the size and shape of the lesion. The value chosen in this work was adapted from a study with human observers within a framework of simulated noisy micrographs [29]. The limit used in this study does not necessarily reflect that which is specific to PET imaging in a low count rate with an iterative reconstruction. Assumptions related to the Rose criterion [27] were probably not completely fulfilled, but the quantitative results that we derived were consistent with the visual analysis. Moreover, an additional analysis (not shown) with a different limit of CNR > 5 did not substantially modify the main conclusions regarding detectability.

The CNR may also be significantly improved using a Gaussian post-filtering with a higher FWHM value to reduce noise in the reconstructed images [26] but at the expense of a more pronounce correlation between voxels. In this work, we have set the post-filtering strength to that used for routine examinations of ^18^ F-FDG considering, firstly, the improvement in the reconstructed spatial resolution provided by the PSF modelling, and secondly to reduce the bias in quantitation for the reconstructed volume.

Regarding the estimation of scattered radiation, it should be noted that it relies on a prior quick analytical reconstruction uncorrected for scattering events [35]. As these preliminary reconstructions are based on very noisy sinograms, we expect as well the scattering simulation may fail to calculate a reliable estimate. Similarly, van Elmbt et al. [19] suggested an additional component of true coincidences affecting the ends of the profile tails of the rebinned sinograms. They assumed that this signal may come from pair production in the LSO crystals by the X-ray bremsstrahlung above 1.022 MeV. This component may have an impact on the scaling of the scattered sinogram to the emission sinogram. This uniform background may also affect the random correction process, but no specific correction was applied to account for.

Finally, we used a single acquisition for the extraction of figures of merit used in this work. The use of multiple independent acquisitions for each of the measuring points would have certainly strengthened the statistical robustness of each measurement, especially when the radioactive concentration in the spheres was less than 1 MBq mL^−1^. We reasonably assume that the use of experimental replicates does not change the basic conclusions of this work.

## Conclusions

This study assessed the MDA for a 30-min ^90^Y-PET imaging session and the accuracy of reconstructed activity concentration according to lesion size and its concentration. The benefit of using TOF information is discussed and shown for concentrations below 2 MBq mL^−1^ or small lesion size. An activity concentration below 1 MBq mL^−1^ may be detected but with a variable quantitative accuracy depending on the lesion size and reconstruction parameters. ^90^Y-PET imaging is probably not feasible for most RAIT procedures due to the very low uptake by the lesions during this type of treatment.

However, the utility of ^90^Y-PET imaging after SIRT in hepatic tumors was demonstrated and could be an opportunity to retrospectively check the distribution of microspheres in the liver and the tumor, and to accurately compute the absorbed dose.

Finally, future developments will be dedicated to a better understanding of the physics and imaging properties of ^90^Y image and data, a further specialization of the acquisition protocols, and derive adequate correction schemes.

## Competing interests

The authors declare that they have no competing interests.

## Authors’ contributions

TC performed the data acquisition, analysis, and interpretation. TC is the main author of the manuscript. TE and EG contributed significantly to the patient study and reviewed and approved the final content of the manuscript. CBM, CA, CR, and LF contributed to the intellectual content (supervision) and critical review of the manuscript. JB and FS interpreted the results, gave a critical review of the work, and contributed to the enhancement of the manuscript. FKB participated to the study design and coordination, and helped draft the manuscript. All authors read and approved the final manuscript.
